# Identifying Premium-Quality Beef in the United States—A Comparison of Beef Palatability from Grain-Finished Young and Mature Beef Cattle with Varying Marbling Scores

**DOI:** 10.3390/foods14040676

**Published:** 2025-02-17

**Authors:** Taylor B. McKinzie, Andrea J. Garmyn, Conner C. McKinzie, Mohammad Koohmaraie, Jerrad F. Legako, Dale R. Woerner, Mark F. Miller

**Affiliations:** 1 Department of Animal and Food Sciences, Texas Tech University, Lubbock, TX 79409, USA; taylor.schertz@ttu.edu (T.B.M.); c.mckinzie11@gmail.com (C.C.M.); jerrad.legako@ttu.edu (J.F.L.); dale.woerner@ttu.edu (D.R.W.); mfmrraider@aol.com (M.F.M.); 2 Department of Food Sciences and Human Nutrition, Michigan State University, East Lansing, MI 48824, USA; 3 Department of Animal Science, Michigan State University, East Lansing, MI 48824, USA; 4 IEH Laboratories and Consulting Group, Lake Forest Park, WA 98155, USA; mk@iehinc.com

**Keywords:** beef, consumer, marbling, old maturity, young maturity

## Abstract

The study objective was to compare the palatability of beef strip loin steaks from young and mature grain-finished cattle across a range of marbling scores. Grain-finished beef carcasses were selected from two maturity groups: old maturity (O; >30 months of age) and young maturity (Y; <30 months of age). Within maturity groups, carcasses were selected to represent five marbling degrees—slightly abundant or greater (SLAB), moderate (MD), modest (MT), small (SM), and slight (SL)—resulting in ten treatment groups. *Longissimus dorsi* samples were removed on day 2 postmortem and cut into 2.5 cm thick steaks for slice shear force measurement, consumer palatability analysis, and proximate analysis. Tenderness, juiciness, flavor liking and intensity, overall liking, acceptability, and willingness to pay were all affected (*p* < 0.01) by treatment group. Palatability ratings generally decreased as marbling score decreased, but adjacent marbling scores often produced similar eating quality. Maturity had the most apparent impact on tenderness, as Y vs. O maturity samples scored greater (*p* < 0.05) for tenderness within four of the marbling scores (SLAB, MD, MT, and SL). Maturity had limited to no impact on juiciness, flavor intensity, and flavor liking. SLAB-Y and MD-Y were more liked overall compared to their O counterparts, but no other maturity differences were noted for overall liking within marbling scores. Grain-finished mature cull cows generated a similar or slightly reduced eating experience to young beef, but greater marbling is required to offset tenderness differences in mature beef.

## 1. Introduction

The global demand for beef has increased over the past 50 years and will continue to increase as the world population reaches 10 billion people by 2050 [1]. Producers and beef processors are tasked with the development and implementation of new strategies to meet the growing demand for red-meat protein. With a focus on beef sustainability, the use of mature cull cows in beef production is a unique opportunity to add value to underused products and could provide a new, high-quality option for consumers in retail and foodservice. Mature cows are culled from the herd for assorted reasons, including difficulty rebreeding, advanced age, genetic improvement, and poor performance or health [2]. Approximately 3.5 million beef cull cows were slaughtered in 2021, representing 10.7% of all cattle slaughtered at federally inspected facilities in the United States [3], with most of the beef produced allocated for ground products. Identifying premium beef from within the cull cow population could add value to this segment if boneless subprimals could be marketed rather than being directed to ground beef production.

Grain-feeding cattle for at least 100 days can improve the carcass merit of cull cows [4,5,6] and improve consumer eating satisfaction in young beef [7,8]. Gredell et al. [9] showed grain-finishing mature cattle improved palatability compared to unfed mature beef and young beef with similar marbling scores. There is value in feeding cull cows prior to slaughter as it elevates the eating quality of top loin steaks, producing a comparable eating experience to that of beef from young cattle with similar marbling scores [10]. Not only does this highlight the economic value by further demonstrating the potential to market certain wholesale cuts from cull cows in retail and foodservice [11,12], but it also shows an improvement in sustainability by fully utilizing the beef from cull cows in unique ways other than simply for ground beef production.

Previous research has shown that USDA carcass maturity does not effectively identify age-related differences in *longissimus* palatability traits from fed beef cattle [10,13,14]. Yet, carcass maturity or dentition often disqualifies highly marbled carcasses from cattle over thirty months of age from USDA-certified programs and the associated premiums, as most programs have a maturity specification that animals are either under 30 months of age or possess either A or B carcass maturity. Additionally, Semler et al. [14] reported that dental age impacted some shear force measurements and sensory tenderness of *longissimus* steaks with slight marbling, but not steaks with small marbling or greater. Given these results, our objective was to examine consumer palatability of beef from young and mature animals, as determined by both dentition and skeletal maturity across a broad range of marbling scores from slight to moderately abundant. We hypothesize eating quality will be comparable between young and old beef within marbling categories, potentially providing support for the inclusion of advanced-maturity beef in premium programs.

## 2. Materials and Methods

### 2.1. Product Collection

Beef carcasses were selected from a single commercial abattoir at 2 days postmortem to represent two maturity groups determined via dentition and skeletal maturity. Old-maturity (O; *n* = 448) beef carcasses were greater than 30 months of age, and young-maturity (Y; *n* = 423) carcasses were less than 30 months of age. Old-maturity carcasses also exhibited advanced skeletal maturity characteristics as they possessed over 35% ossification in the first five cartilaginous buttons of the thoracic vertebrae, indicating the chronological ages of the mature cattle were 42 months and older [15]. Within maturity groups, carcasses were selected to represent five marbling categories: slightly abundant or greater (SLAB), moderate (MD), modest (MT), small (SM), and slight (SL). The distribution of carcasses by marbling and maturity can be found in Table 1.

Cattle in the study were commercially finished, receiving supplemental energy from a grain-finished diet before harvest [9,10,16]. Feeding duration and ration was unknown due to their selection from a commercial facility; however, “White Cow” programs in the Northern Plains (Nebraska), where the cattle were sourced for this particular abattoir, are known to feed high-concentrate rations (corn, soybean, and roughage) for approximately 140 days [10,17]. Carcass data were collected by trained personnel from Texas Tech University at the time of selection, including fat thickness, ribeye area, and hot carcass weight. Marbling score data were collected via the VBG2000 (e+v Technology GmbH & Co., KG, Oranienburg, Germany) grading camera with an LED.

### 2.2. Steak Fabrication

On day 2 postmortem, *longissimus lumborum* sections measuring ~10 cm were collected from the right side of each beef carcass from the anterior-most portion of the strip loin by trained personnel from Texas Tech University. Each section was denuded, removing all subcutaneous fat, accessory muscles, and intermuscular fat. Sections were portioned into 2.5 cm thick steaks, and steaks were assigned to the following analyses: slice shear force (SSF) measurement, consumer palatability analysis, and proximate analysis. Slice shear force analysis was conducted on collection day, and the remaining steak samples were individually vacuum-packaged, stored at 0 to 4 °C, and frozen (−10 °C) on day 21 postmortem. Frozen steaks were later shipped to the Texas Tech University Gordon W. Davis Meat Laboratory (Lubbock, TX, USA) for further analyses.

### 2.3. Slice Shear Force (SSF)

Slice shear force analysis was conducted on steaks 2 days postmortem to provide an early indication of tenderness. This early measure of tenderness could serve as a sortation tool, when used in conjunction with carcass characteristics, to identify carcasses with potentially exceptional palatability. Steaks were cooked on a clamshell grill (George Foreman, Spectrum Brands, Houston, TX, USA) to an internal temperature of 71 °C. After a 3 min rest, the final internal temperature was recorded for each sample using a digital thermometer (Digi-Sense Type J, Cole-Parmer Instrument Company, Vernon Hills, IL, USA). The American Meat Science Association [18] SSF protocol was used to produce a 1 cm thick sample per steak. The 1 cm thick sample was placed on the SSF machine (G-R Electric Manufacturing Company LLC, Manhattan, KS, USA), with the muscle fibers running perpendicular to the blade. Values for SSF were recorded for each sample. Using the American Society for Testing and Materials (ASTM) guidelines, steaks were categorized as “Certified Tender” when SSF values were 20.0 kg or less or “Certified Very Tender” for steaks with SSF values of 15.4 kg or less.

### 2.4. Proximate Analysis

Proximate analysis was performed to determine the chemical composition (percentage of fat, moisture, and protein) for each sample. Steaks were thawed in a refrigerator at 2 to 4 °C for 24 h. Each sample was ground twice (coarse and fine) using a tabletop grinder (Cabela’s Deluxe Meat Grinder, Cabela’s Inc., Sidney, NE, USA). Proximate analysis was conducted using an AOAC-approved (Official Method 2007.04) near-infrared spectrophotometer (FoodScan, FOSS NIRsystems, Inc., Laurel, MD, USA).

### 2.5. Consumer Panels

The Texas Tech University Institutional Review Board approved all procedures using human subjects in this study (IRB#: IRB2021-799; approved 10 November, 2021). Consumers (*n* = 320; 20 consumers/session) were recruited to participate in 16 sessions at the Texas Tech University Animal Science Building (Lubbock, TX, USA). Consumers were only allowed to participate once and had to meet two specific criteria: favor a medium degree of doneness and regularly consume beef. An electronic ballot was developed using Qualtrics (Qualtrics, Provo, UT, USA) and provided to consumers on tablets (Apple iPad, Apple, Cupertino, CA, USA). Consumers randomly selected a booth and were provided an iPad, water, diluted apple juice, an empty cup, unsalted crackers, and eating utensils. Consumers were tasked to fill out a demographic sheet containing questions related to gender, age, household size, marital status, ethnic origin, annual household income, highest level of education completed, consumption of beef, and degree of doneness preference.

For each session, 50 predetermined steaks (5 representing each treatment group) were thawed in a refrigerator at 2 to 4 °C for 24 h before cooking for consumer panels. Each steak was cut in half (horizontally) before being placed on a grill to accommodate the number of participants in each session. Steaks were cooked on clamshell grills (George Foreman, Spectrum Brands, Houston, TX, USA) to a final internal temperature of 68 to 71 °C, indicating a medium degree of doneness. The final temperature was taken with a digital thermometer (Thermapen ONE, ThermoWorks, American Fork, UT, USA) and recorded. Each sample (steak half) was portioned into two equally sized pieces and plated to be served to two predetermined consumers.

A 10 × 10 Latin Square was used to generate a randomized serving order. Consumers received 10 steak samples representing all ten treatment groups representing marbling score (SLAB, MD, MT, SM, and SL) and maturity combinations (O and Y maturity) to evaluate tenderness, juiciness, beef flavor intensity, flavor liking, and overall liking using 100 mm anchored line-scales (0 = extremely tough/dry/bland/dislike extremely; 100 = extremely tender/juicy/intense/like extremely). Acceptability (yes/no) for overall liking, tenderness, juiciness, and flavor liking was assessed for each sample. Additionally, willingness to pay (WTP; USD/0.45 kg) was collected on an individual-sample basis. Each panel took 90 min to complete, and consumers were provided a monetary stipend.

### 2.6. Statistical Analysis

All data were analyzed using R statistical software, version 4.1.2 (2021), and significance level was set at α = 0.05 for all analyses. Least squares means were calculated for all response variables in the study by using the lm() function from base R to fit a linear model with treatment group (combination of maturity and marbling) as the fixed effect in a completely randomized model. Analysis of variance (ANOVA) was computed by using the anova() function from the lmerTest package [19]. The emmeans package allowed comparisons to be tested for significance with Tukey-adjusted pairwise and significance at α = 0.05 [20]. The prop.table() function determined the probability of acceptable and unacceptable responses for consumer panels. Pearson correlation coefficients were computed and analyzed for significance between consumer palatability traits, proximate composition, SSF, and carcass characteristics through the cor.test() function. The primary objective of analyzing Pearson correlation coefficients was to test for the relationship between paired samples, and only significant (*p* < 0.01) Pearson correlations were noted.

## 3. Results

### 3.1. Carcass Characteristics

The effects of treatment group on the least squares means of carcass characteristics are shown in Table 1. Marbling score was different between treatment groups (*p* < 0.01), decreasing from SLAB to SL. Expectedly, within each marbling degree, O and Y were similar (*p* > 0.05), with the exception of SL, where SL-Y had a greater amount of marbling than SL-O (*p* < 0.01).

Treatment group impacted (*p* < 0.01) hot carcass weight, fat thickness, and ribeye area. SLAB-O had the heaviest carcasses (*p* < 0.05) and measurements were similar (*p* > 0.05) to SLAB-Y, MD-O, MT-Y, and MT-O. The SM-Y group included the lightest carcasses (*p* < 0.05), and measurements did not differ from SL-Y and SL-O (*p* > 0.05). Overall, hot carcass weights generally decreased as marbling score decreased in O fed beef carcasses. Alternatively, Y fed beef carcasses did not exhibit the same trend, as MT-Y had the heaviest average hot carcass weight.

Beef carcasses were generally fatter at the 12th rib measurement as marbling score increased within both carcass maturity groups (*p* < 0.01). SLAB and MD carcasses had greater (*p* < 0.05) fat thickness than other marbling scores, regardless of maturity. Expectedly, SL-O and SL-Y had less fat opposite the 12th rib (*p* < 0.05) compared to other treatment groups but were similar to MT-O, SM-Y, and SM-O (*p* > 0.05). SL-Y beef carcasses expressed a larger ribeye area (*p* < 0.05) than all O-maturity carcasses and were comparable to SLAB-Y, MD-Y, MT-Y, and SM-Y (*p* > 0.05).

### 3.2. Slice Shear Force with Certified Tender and Very Tender Passage Rates

Slice shear force values and percentages classified as Certified Tender and Very Tender are presented in Table 2. Treatment group influenced (*p* < 0.01) slice shear force, where SLAB-Y required less force (*p* < 0.05) to shear than MD-O, MT-O, SM-O, and SL-O; SSF values were similar for all Y samples and SLAB-O (*p* > 0.05). Likewise, SSF values did not differ (*p* > 0.05) among O samples. MD-O and MT-O had greater (*p* < 0.05) SSF values than their Y counterparts; otherwise, SSF values were similar (*p* > 0.05) within marbling scores.

On day 2 postmortem, beef steak samples with SSF values less than 20.0 kg qualified for a Certified Tender designation, and beef steak samples shearing less than 15.4 kg qualified for a Certified Very Tender classification. Few differences were noted between Y and O samples within a marbling score for percent classified as Certified Tender, as SLAB-Y, MD-Y, MT-Y, SLAB-O, MD-O, and MT-O were similar (*p* > 0.05), along with SM-Y and SL-O. However, a greater (*p* < 0.05) percentage of SM-Y samples were deemed Tender compared to SM-O, but more SL-O samples were considered Tender compared to SL-Y. Maturity had a greater impact on the classification as Very Tender, with a greater (*p* < 0.05) percentage of SLAB, MD, and MT steaks from Y carcasses being categorized as Very Tender compared to their O counterparts. The percentage of Very Tender samples did not differ (*p* > 0.05) between Y and O samples within MT and SL marbling scores.

### 3.3. Proximate Analysis

Proximate analysis was used to evaluate the chemical percentages of fat, moisture, and protein present in each sample (Table 3). Treatment group impacted the percentage of fat and moisture (*p* < 0.01). Fat percentage decreased as the marbling score decreased within each maturity. MD-Y had greater (*p* < 0.05) fat percentage than MD-O; otherwise, fat percentage was similar (*p* > 0.05) within each marbling score when comparing Y and O. Moisture percentages decreased as fat percentages increased from SLAB to SL. Protein percentages did not vary (*p* = 0.17) across treatment groups.

### 3.4. Consumer Demographics

Demographic profiles and meat consumption responses of consumers (n = 320) that participated in consumer palatability testing from Lubbock, TX, are presented in Table 4. The majority of consumers were female (55%) and identified as Caucasian/white (75.6%). The second most common ethnic origin was Hispanic/Latin American (11.6%). Age was evenly distributed between age groups. Gender demographics were slightly different from the United States (U.S.) Census Bureau [21] population statistics as females represent 51% and males represent 49% of the U.S. population. The ethnic origin demographics were similar to the U.S. Census Bureau report with 75.8% Caucasian/White; however, the Hispanic/Latin American and African American ethnicities represent 18.9% and 13.6% of the U.S. population, respectively. The majority of consumers were married (60%), and the most common household size was 2 people, which aligned with the U.S. Census Bureau report [21]. Additionally, most consumers in the study (36.2%) had an annual household income of over USD 100,000, whereas nearly 80% of consumers had at least some college or technical school experience or were a college graduate. Over 95% of participants consume beef weekly, with almost a third of panelists indicating they consume beef daily. The majority of consumers preferred a medium-rare degree of doneness for beef (45.6%).

### 3.5. Consumer Palatability Evaluation

Palatability data collected from consumers (n = 320) in Lubbock, TX, are presented in Table 5. Tenderness, juiciness, flavor intensity, flavor liking, and overall liking were influenced (*p* < 0.01) by treatment group. Palatability ratings generally increased as marbling increased, but adjacent marbling scores often produced similar eating quality results.

Tenderness was different (*p* < 0.01) across treatment groups (Table 5), with tenderness generally decreasing as marbling score decreased. This trend was observed for both Y and O maturity. The SLAB-Y was more tender (*p* < 0.05) than all other treatment groups except MD-Y (*p* > 0.05). SL-O, SM-O, and MT-O were among the least tender according to consumers. Maturity had the most apparent impact on tenderness, of the palatability traits, as tenderness was scored greater (*p* < 0.05) for Y- vs. O-maturity samples within four of the marbling scores (SLAB, MD, MT, and SL).

Juiciness was different (*p* < 0.01) across treatment groups (Table 5). Juiciness decreased as marbling decreased, but again, there were some similarities in juiciness scores between adjacent marbling scores. Maturity had little impact on juiciness, as juiciness was only scored greater (*p* < 0.05) for Y- vs. O-maturity samples within the MD marbling score. Juiciness was similar (*p* > 0.05) between Y and O samples for all other marbling scores. The SLAB-Y, MD-Y, and SLAB-O were not different (*p* > 0.05) and rated juicier than other treatment groups, followed by MT-Y and MD-O. The SL-O samples were less juicy (*p* < 0.01) than other treatment groups but did not differ from SL-Y, SM-O, and SM-Y (*p* > 0.05).

Treatment group influenced (*p* < 0.01) flavor intensity and flavor liking, as shown in Table 5. As marbling decreased, the flavor intensity and flavor liking decreased. Flavor liking and beef flavor intensity were similar (*p* > 0.05) between Y and O samples for all marbling scores, suggesting maturity had minimal influence on flavor. The SLAB-Y, SLAB-O, and MD-Y were similar (*p* > 0.05) and were considered to have a more (*p* < 0.05) intense beef flavor, which was appreciated more by consumers than the other treatment groups. However, there were similarities in flavor ratings among adjacent marbling scores. For example, MD-Y and MT-Y were similar, and SLAB-O, MD-O, and MT-O did not differ (*p* > 0.05). The SL-O group was less desirable (*p* < 0.05) for flavor intensity and liking than other treatment groups but was similar to MT-O, SM-O, SM-Y, and SL-Y (*p* > 0.05) for flavor intensity and flavor liking.

Overall liking was influenced by treatment group (Table 5). As noted previously for all palatability traits, overall liking decreased as marbling score decreased. The impact of maturity on overall liking was more apparent in the higher marbling scores, as SLAB-Y was more liked than SLAB-O. Likewise, MD-Y was more liked than MD-O. Overall liking was similar (*p* > 0.05) between Y and O samples for all other marbling scores. The SLAB-Y and MD-Y were more liked (*p* < 0.05) overall compared to all other treatment groups, but MD-Y was similar (*p* > 0.05) to SLAB-O. There were no differences between SM-Y, SL-Y, MT-O, SM-O, and SL-O (*p* > 0.05), which scored lowest for overall liking.

### 3.6. Consumer Acceptability

The percentages of samples acceptable for tenderness, juiciness, flavor liking, and overall liking, as well as consumer willingness to pay (WTP), are presented in Table 6. Consumer acceptability for each palatability trait was influenced by treatment group (*p* < 0.01). Much like palatability ratings, acceptability decreased as marbling score decreased. Tenderness acceptability followed the trend of tenderness scores, where a greater (*p* < 0.05) proportion of samples were deemed acceptable for tenderness (*p* < 0.05) for Y- vs. O-maturity samples within each marbling score. Juiciness acceptability was greater (*p* < 0.05) for two of the five marbling scores (MD and MT) when comparing Y samples to their O counterparts. A greater proportion (*p* < 0.05) of samples were considered acceptable for flavor for two of the five marbling scores (SLAB and MD) when comparing Y vs. O samples. Lastly, overall acceptability was greater (*p* < 0.05) for three of the marbling scores (SLAB, MD, and SL) when comparing Y samples to their O counterparts.

Willingness to pay was influenced (*p* < 0.01) by treatment group (Table 6). Consumers were willing to pay more (*p* < 0.051) for SLAB-Y and MD-Y beef steaks compared to all other treatment groups; however, WTP was similar for MD-Y, SLAB-O, MD-O, and MT-Y. Consumers were willing to pay less (*p* < 0.05) for SM and SL beef steaks, regardless of maturity, along with MT-O, which were all similar (*p* > 0.05). Within marbling scores, consumers were willing to pay more (*p* < 0.05) for SLAB-Y and MT-Y compared to their O counterparts. No other differences (*p* > 0.05) were observed in WTP between Y and O samples within a marbling score.

### 3.7. Correlations

Pearson correlation coefficients for consumer palatability traits, proximate composition, SSF, and carcass characteristics are presented in Table 7. Overall liking was strongly correlated with tenderness (r = 0.80), juiciness (r = 0.70), and flavor liking (r = 0.88). With the exception of SSF and percent protein, the consumer sensory evaluation traits were highly correlated (*p* < 0.01) with each proximate component and carcass characteristic. SSF was highly correlated (*p* < 0.01) with percent fat and moisture. Percent protein was not correlated (*p* < 0.01) with any variables.

## 4. Discussion

The results of the current study indicate highly marbled beef from grain-finished mature cull cows possesses similar compositional traits and only slightly reduced eating experiences compared to young, conventional beef. Feeding a high-concentrate finishing ration to mature cull cows pre-harvest has the potential to improve carcass traits, including marbling score, lean maturity score [11,22,23,24], and fat color [25,26], resulting in greater profitability for the producer and packer relative to non-fed mature cull cows [26,27]. With an emphasis on current beef supply and demand, highly marbled beef from long-grain-fed mature cull cows could serve as a highly palatable product for consumers. Furthermore, potential establishment of a branded beef program to market certain muscles from grain-finished mature cull cows is warranted to offer a product comparable in price to high-quality young fed beef, thus benefiting the packer’s margins and providing an affordable, highly marbled option for consumers.

### 4.1. Carcass Characteristics

Grain-finishing mature cull cows typically results in heavier carcasses, with a larger ribeye area, greater fat opposite the 12th rib, and higher degree of marbling relative to non-fed cull cows [22,27], resulting in a higher merchandising value [26,27]. When compared to A-maturity cattle, mature cull cows can produce carcasses with comparable carcass characteristics. Overall, carcass characteristics within marbling scores were similar between Y and O carcasses, with the exception of SL, where SL-Y had a higher degree of marbling and a larger ribeye area.

### 4.2. Slice Shear Force with Certified Tender and Very Tender Passage Rates

SSF values were comparable between Y and O steaks within marbling scores for three of the five marbling scores (SLAB, SM, and SL). Only MD-Y and MT-Y had SSF values lower than their O counterpart. SSF values for all samples were exceptionally tender, with mean values far below Certified Tender and Certified Very Tender thresholds, indicating the harvesting and beef fabrication processes, along with the cooking methods used in the study, were conducive to producing tender beef. Slice shear force analysis was conducted on steaks 2 days postmortem. Despite the limited proteolysis, high percentages of steak samples could be classified as either Tender or Very Tender. Differences existed between treatment groups, more so in the percentage classified as Very Tender. Over 90% of SLAB, MD, and MT samples could be classified as Certified Tender, regardless of maturity. This passage rate for Certified Tender for highly marbled, long-fed mature beef is very promising and could offer the potential for an additional marketing claim and carcass premium if a branded beef program was developed for qualifying carcasses. Cho et al. [28] found no differences in shear force values of *longissimus thoracis* muscles between 30-month-old steers compared to 47- to 53-month-old cull cows. Mean shear force values for steers and cows would qualify for a Certified Very Tender program [28].

It is important to note that SSF was correlated with the percentage of fat and moisture within the samples, rather than tenderness (Table 7). Although SSF is a common method for assessing tenderness, it does not fully represent the tenderness experienced by consumers during consumption. There was no relationship between the subjective SSF values and the objective tenderness scores assigned by consumers. Although SSF has appeared to be more accurate than WBSF with greater repeatability estimates [29], it is believed that SSF is more influenced by intramuscular fat, not by maturity type [9,13,14]. Therefore, beef steaks with the same marbling degree may have similar SSF values; however, consumers could still detect differences in tenderness, possibly because of variations in heat-soluble collagen levels.

Overall, the previous literature has shown that grain-feeding mature cull cows improves shear force values compared to non-fed mature cull cows [9,14,22,24]. Although Cranwell et al. [24] found no difference in shear force values between mature cattle fed for 28 d versus 56 d [24], Cashman et al. [10] found grain-finished mature cull cows fed over 140 days yielded comparable shear force values to beef from young cattle, which aligns with the results of the current study. However, it is important to note that not all mature fed beef programs may yield these SSF results, as this study applies to long-fed mature cull cows.

### 4.3. Proximate Analysis

On average, fat percentage increased as marbling score increased within each maturity group, which is similar to the prior literature [10,30]. These results reinforce the accuracy of the camera technology in the study to accurately assign marbling degrees, as marbling has a direct correlation to the percentage of fat in the *longissimus dorsi* muscle. Fat and moisture percentages have an inverse relationship [9,10,31,32]; therefore, moisture percentages decreased as fat percentages increased from SL to SLAB. There were no differences between treatment groups for protein percentage. The findings for percent protein in the samples contrasted those of Gredell et al. [9], who concluded that protein increased as fat increased.

### 4.4. Consumer Palatability, Acceptability, and Willingness to Pay

Results of the current study are consistent with the previous literature as beef palatability traits, including tenderness, juiciness, and flavor liking, improved as marbling scores increased [9,31,33,34]. Treatment in the current study influenced all beef palatability attributes for consumer ratings and acceptability (*p* < 0.01). Furthermore, tenderness, juiciness, flavor liking, and overall liking were all positively correlated with both marbling score and fat percentage. However, Pearson correlation coefficients are limited to comparisons between two traits and only indicate the presence of linear relationships. Determining fatty acid profiles in future studies could help better define the relationship between fat and palatability. However, consumers rated SLAB-Y and MD-Y the most tender (*p* < 0.01), coinciding with the previous literature noting the positive relationship between marbling and improved consumer tenderness perception in A-maturity beef [30,31,34]. Cho et al. [28] found no differences in palatability attributes when comparing beef from young steers and mature cull cows; however, consumer tenderness ratings in this study more closely align with the previous literature [10,26,27], where beef from A-maturity carcasses was rated more tender than beef from C-, D-, and E-maturity cull cow carcasses. Hilton et al. [26] identified 30 to 40% of the tenderness variation observed was related to the interaction of marbling and maturity in A-, B-, C-, D-, and E-maturity beef carcasses, where A maturity was the most tender and E maturity was the toughest. When analyzing overall tenderness comparing six different marbling scores, results showed tenderness improved when marbling increased [26], which agrees with the findings of this study where SLAB was the most tender, followed by MD, MT, SM, and SL for O and Y carcass types. Consumer palatability work by Stelzleni et al. [27] found A-maturity beef with slight marbling was rated as “slightly tender”, while beef from grain-finished mature cull cows was rated as “slightly tough”. However, it is unclear how many days the mature cull cows were placed on feed before harvest [27]. Days on feed for cull cows could influence results, as trends in the previous literature show a direct relationship between tenderness and days on feed [23,24]. Mature cull cows in the present study were placed on a high-energy, grain-finishing diet for a minimum of 140 days pre-harvest. Consumer tenderness ratings and acceptability were numerically higher than consumer tenderness scores reported by Cashman et al. [10], but trends for greater tenderness scores for Y versus O samples within marbling scores were consistent between the two studies.

Tenderness was an important factor to measure, via SSF and untrained panelists, as beef carcasses from cattle over thirty months of age can produce tougher steaks because of mature collagen crosslinks [10,35]. SSF values indicated SLAB-O strip loin steaks sheared similarly to all treatment groups associated with young beef carcasses; however, tenderness differences were evident in consumer palatability work, where SLAB-O was less tender than SLAB-Y and MD-Y. With the use of Warner Bratzler Shear Force (WBSF), Cashman et al. [10] noted minimal differences within marbling scores when comparing O and Y samples. In contrast, consumer tenderness values differed between young and mature samples within most marbling scores [10]. Studies have shown sensory panel tenderness ratings should correlate with shear force when using *longissimus dorsi* [36,37]; however, the variation present in Cashman et al. [10] and the current study could be related to the variation in heat-soluble collagen present in mature cull cows [9]. As an animal matures, the amount of heat-soluble collagen decreases in the muscle and is replaced with insoluble collagen [27,38,39,40], resulting in a significantly tougher product. Alternatively, the placement of mature cull cows on a grain-based diet prior to slaughter has been noted in the previous literature to reinforce the amount of heat-soluble collagen present in the muscle, which would increase the tenderness, because of protein synthesis [22,24,27]. Miller et al. [22] found that the use of a high-energy diet rather than a low-energy diet during the finishing phase of cull cows could improve tenderness by increasing the proportion of heat-soluble collagen versus insoluble collagen present in the muscle. Analysis of heat-soluble collagen levels within each treatment group would be beneficial to better understand the ratio of insoluble to heat-soluble portions of the total collagen.

Maturity had no effect on juiciness within each marbling score, with the exception of MD-Y, which was greater for juiciness compared to MD-O (*p* < 0.01). Moreover, consumers found no differences in flavor intensity and flavor liking between Y- and O-maturity carcasses within marbling scores. Interestingly, consumers in Cashman et al.’s study [10] rated SLAB+ beef samples from mature fed cows less flavorful compared to SLAB+ and MD/MT beef from young fed cattle, but more acceptable for flavor liking. It is important to note that the marbling scores between young and mature SLAB+ in Cashman et al.’s study [10] were different, which could have influenced the differences in flavor liking. Additionally, steaks were cooked using a char broiler [10], which has been noted to produce greater flavor liking scores and acceptability, while potentially masking off-flavors, in comparison to a clamshell grill [41]. Previous studies have shown mature cull cows can generate more off-flavors, such as painty, fishy, or grassy [26,27]. Although off-flavors were not analyzed in the present study, a clamshell grill was used to avoid the potential masking of undesirable off-notes for consumers, thus removing outside cooking influences. Nonetheless, consumer flavor liking scores were greater in the current study for Y and O carcasses within each marbling group compared to those found by Cashman et al. [10].

Carcass maturity had no effect on overall liking for MT, SM, and SL marbling scores; however, differences were evident amongst the highly marbled treatment groups (SLAB and MD). Tenderness variation between Y and O samples seemingly contributed most to the variation in overall liking, given the limited impact of the maturity on flavor and juiciness. For years, tenderness has been regarded as the most important palatability trait influencing the consumers overall perception of beef [23,33,42]. Overall liking scores and acceptability were numerically higher across treatment groups compared to Cashman et al. [10]; however, there was a larger spread between SLAB-Y and SLAB-O in the present study, which could be related to tenderness. Intramuscular fat in beef steak samples had an influence on consumer palatability [9,31,33,34]. Overall, consumer acceptability for beef palatability factors decreased as the marbling score decreased, but maturity factored into acceptance as well. This trend aligns with the beef industry’s emphasis on marbling as a key indicator for a positive eating experience. In Europe, Liu et al. [43] highlighted that flavor liking has the greatest influence on consumer overall liking. Therefore, overall liking scores for Y and O beef may become closer depending upon the background and region of the consumers.

Based upon the previous literature and data collected in this study, highly marbled beef from grain-finished mature cull cows offers a palatable product for consumers [10,26,27], and consumers are willing to pay more for beef that is considered to be more tender, juicy, and flavorful. Unlike previous studies, this analysis is the first of its kind to gauge consumers’ willingness to pay for grain-fed mature cull cow beef compared to conventional, A-maturity beef. Maturity reduced WTP in some instances; however, it is important to note the value difference in SLAB-O and MD-O compared to MT-Y, SM-Y, and SL-Y. Currently, highly marbled steaks from mature cull cows are being marketed at a dollar value far less than Top Choice, Low Choice, and Select steaks in the retail case. Overall, consumers indicated they would be willing to pay similar prices for SLAB-O and MD-O compared to Top Choice beef. Additionally, consumers were willing to pay between 0.60 USD/lb and 1.50 USD/lb more, on average, for SLAB-O compared to MT-Y, SM-Y, and SL-Y. Furthermore, consumers were willing to pay between 0.30 USD/lb and 1.20 USD/lb more for MD-O compared to MT-Y, SM-Y, and SL-Y. This finding highlights that SLAB-O and MD-O beef, at least top loin steaks, is being tremendously undervalued. The National Weekly Direct Slaughter Cattle report showed carcasses identified as a hard bone (those with advanced skeletal maturity at grading) are heavily discounted (42 USD/hundred weight), while carcasses that are deemed over thirty months of age based on dentition are also discounted (14 USD/hundred weight), but these discounts vary throughout the year [44]. Moreover, beef carcasses must be under 30 months of age to qualify for most USDA-certified or -branded programs, which typically require at least MT marbling to qualify. These programs are often accompanied by premiums [45,46]. The O carcasses in the current study could be subject to carcass discounts but also cannot be awarded premiums even though minimum marbling requirements were met. Consumers in the current study were willing to pay the most for SLAB-Y, but consumers were willing to pay comparable prices for SLAB-O to those they paid for MD-Y and MT-Y, which were likely eligible for a premium branded program and would garner premiums at both the carcass and retail level. At the retail level, the price of a boneless top loin steak averages about 15.99 USD/lb in a Branded Beef Program, 10.99 USD/lb for a USDA Low Choice product, and 7.99 USD/lb for a USDA Select product [46]. On the other hand, the price of a boneless top loin steak from a non-graded carcass, including mature cull cows, can range from 3.99 USD/lb to 6.99 USD/lb [46]. Beef from grain-finished mature cull cows has significant unrealized gains. Top loin steaks from highly marbled mature beef carcasses could be marketed at a price point that is more affordable than USDA Prime, yet more valuable than the current market price. Therefore, a potential branded beef program for these beef carcasses could be investigated.

## 5. Conclusions

Maturity had the most apparent impact on tenderness, with limited to no effect on juiciness, flavor intensity, and flavor liking. Overall, long-fed grain-finishing of mature cull cows offered a similar eating experience to young beef when highly marbled, but greater marbling was required to offset tenderness differences in mature beef. These findings reinforce the positive influence of marbling on consumer eating experiences. Additionally, values for consumer willingness to pay indicated top loin steaks from highly marbled mature beef carcasses could be marketed at a price point that is more affordable than USDA Prime yet more valuable than the current market price. Therefore, a potential branded beef program for long-fed mature cull cows could be investigated. It should be noted that not all long-fed grain-finished mature cull cows will respond the same. In fact, this phenomenon allowed us to collect samples from carcasses with a range of marbling scores. Additionally, results may vary at different abattoirs or with consumers in other regions within the US or around the globe. Therefore, results must be interpreted with consideration to these factors. Further research analyzing insoluble versus heat-soluble collagen between O and Y beef strip steak samples could better demonstrate the impact physiological age maturity has on beef products. Additionally, nutrient profiling in future studies could help differentiate flavor differences.

## Figures and Tables

**Table 1:** The effect of treatment group on selected carcass characteristics from beef carcasses with varying marbling degrees ^1^ from young (Y) ^2^ and old (O) ^3^ grain-fed beef carcasses.

Treatment	n	Marbling ^4^ Scores	Hot Carcass Weight, kg	Fat Thickness, cm	Ribeye Area, cm^2^
SLAB-Y	85	756 ^a^	431 ^ab^	1.83 ^a^	84.5 ^ab^
MD-Y	84	655 ^b^	424 ^bc^	1.58 ^ab^	86.0 ^ab^
MT-Y	84	550 ^c^	432 ^ab^	1.41 ^bc^	87.4 ^ab^
SM-Y	85	448 ^d^	383 ^e^	1.18 ^cd^	85.1 ^ab^
SL-Y	85	362 ^e^	394 ^de^	0.97 ^d^	92.4 ^a^
SLAB-O	89	757 ^a^	454 ^a^	1.56 ^ab^	77.4 ^b^
MD-O	90	649 ^b^	437 ^ab^	1.58 ^ab^	78.6 ^b^
MT-O	91	549 ^c^	431 ^ab^	1.28 ^bcd^	78.4 ^b^
SM-O	88	442 ^d^	412 ^bcd^	1.22 ^cd^	78.4 ^b^
SL-O	90	340 ^f^	397 ^cde^	0.96 ^d^	77.5 ^b^
SEM ^5^		3.2	6.4	0.1	2.8
*p*-value		<0.01	<0.01	<0.01	<0.01

^a–f^ Within a column, estimated marginal means without a common superscript differ (*p* < 0.05). ^1^ Marbling degrees: SLAB = ≥slightly abundant^00^; MD = moderate^00^ to moderate^100^; MT = modest^00^ to modest^100^; SM = small^00^ to small^100^; SL = slight^00^ to slight^100^. ^2^ Young (Y): beef carcasses from grain-fed cattle < 30 months of age (A skeletal maturity). ^3^ Old (O): beef carcasses from grain-fed cattle > 30 months of age (C, D, and E skeletal maturity). ^4^ Marbling scores: 300 = slight; 400 = small; 500 = modest; 600 = moderate; 700 = slightly abundant; 800 = moderately abundant (collected by plant camera; VBG2000 with LED). ^5^ SEM is the (largest) pooled standard error of least squares means.

**Table 2:** The effect of treatment group on slice shear force (SSF) and percentage classified as Certified Tender (SSF < 20.0 kg) and Very Tender (SSF < 15.4 kg) at two days postmortem for young (Y) ^1^ and old (O) ^2^ grain-fed beef carcasses with varying marbling degrees ^3^.

Treatment	n	SSF, kg	Tender, %	Very Tender, %
SLAB-Y	85	11.2 ^d^	98.8 ^a^	88.2 ^a^
MD-Y	84	11.6 ^cd^	97.6 ^a^	88.1 ^a^
MT-Y	84	12.3 ^bcd^	96.4 ^ab^	71.4 ^bcd^
SM-Y	85	12.8 ^abcd^	95.3 ^ab^	78.8 ^ab^
SL-Y	85	12.7 ^abcd^	89.4 ^bc^	75.3 ^bc^
SLAB-O	89	13.1 ^abcd^	97.8 ^a^	75.3 ^bc^
MD-O	90	13.8 ^ab^	92.2 ^abc^	73.3 ^bc^
MT-O	91	14.6 ^a^	91.2 ^abc^	57.1 ^d^
SM-O	88	14.5 ^a^	83.0 ^c^	64.8 ^cd^
SL-O	90	13.6 ^abc^	93.3 ^ab^	72.2 ^bc^
SEM ^4^		0.4	3.0	5.1
*p*-value		<0.01	<0.01	<0.01

^a–d^ Within a column, estimated marginal means without a common superscript differ (*p* < 0.05). ^1^ Young: beef carcasses from grain-fed cattle < 30 months of age (A skeletal maturity). ^2^ Old: beef carcasses from grain-fed cattle > 30 months of age (C, D, and E skeletal maturity). ^3^ Marbling degrees: SLAB = ≥slightly abundant^00^; MD = moderate^00^ to moderate^100^; MT = modest^00^ to modest^100^; SM = small^00^ to small^100^; SL = slight^00^ to slight^100^. ^4^ SEM is the (largest) pooled standard error of least squares means.

**Table 3:** The effect of treatment group on percentage of chemical fat, moisture, and protein for beef strip steaks from young (Y) ^1^ and old (O) ^2^ grain-fed beef carcasses with varying marbling degrees ^3^ determined by proximate analysis.

Treatment	n	Fat, %	Moisture, %	Protein, %
SLAB-Y	43	11.52 ^a^	63.9 ^g^	24.3
MD-Y	45	9.48 ^b^	65.0 ^f^	24.4
MT-Y	46	7.19 ^cd^	66.6 ^de^	25.2
SM-Y	40	5.26 ^ef^	68.1 ^bc^	25.8
SL-Y	42	3.60 ^g^	69.2 ^a^	31.3
SLAB-O	40	11.45 ^a^	64.0 ^g^	24.4
MD-O	40	7.95 ^c^	66.2 ^e^	25.2
MT-O	41	6.27 ^de^	67.4 ^cd^	25.7
SM-O	41	4.52 ^fg^	68.8 ^ab^	25.7
SL-O	40	3.51 ^g^	69.6 ^a^	26.0
SEM ^4^		0.38	0.30	2.27
*p*-value		<0.01	<0.01	0.17

^a–g^ Within a column, estimated marginal means without a common superscript differ (*p* < 0.05). ^1^ Young: beef carcasses from grain-fed cattle < 30 months of age (A skeletal maturity). ^2^ Old: beef carcasses from grain-fed cattle > 30 months of age (C, D, and E skeletal maturity). ^3^ Marbling degrees: SLAB = ≥slightly abundant^00^; MD = moderate^00^ to moderate^100^; MT = modest^00^ to modest^100^; SM = small^00^ to small^100^; SL = slight^00^ to slight^100^. ^4^ SEM is the (largest) pooled standard error of least squares means.

**Table 4:** Demographic characteristics and meat consumption responses of consumers (n = 320) who participated in consumer palatability panels in Lubbock, TX, USA.

Characteristics	Response	Frequency, %
Sex	Male	45.0
	Female	55.0
Age	Under 20	10.3
	20 to 29 years old	20.3
	30 to 39 years old	16.6
	40 to 49 years old	20.6
	50 to 59 years old	15.3
	Over 60	16.9
Household Size	1 person	10.9
	2 people	29.1
	3 people	17.8
	4 people	21.6
	5 people	12.8
	6 people	6.6
	>6 people	1.3
Marital Status	Single	40.0
	Married	60.0
Ethnic Origin	African American	8.8
	Asian	0.6
	Caucasian/White	75.6
	Hispanic/Latin American	11.6
	Mixed Race (2 or more of above)	2.8
	Other	0.6
Annual Household Income	<USD 20,000	15.9
	USD 20,000–USD 50,000	15.0
	USD 50,001–USD 75,000	17.2
	USD 75,001–USD 100,000	15.6
	>USD 100,000	36.2
Education	Non-High School Graduate	2.2
	High School Graduate	19.1
	Some College/Technical School	33.1
	College Graduate	29.4
	Post-College Graduate	16.2
Average Beef Consumption	Daily	19.4
	4 to 5 times per week	30.3
	2 to 3 times per week	31.9
	Weekly	13.8
	Every other week	0.9
	Monthly	2.2
	Rarely	1.6
Degree of Doneness	Blue Rare	0.9
	Rare	5.6
	Medium-Rare	45.6
	Medium	25.3
	Medium-Well	16.6
	Well-Done	5.9

**Table 5:** The effect of treatment group on consumer (n = 320) sensory scores ^1^ for palatability traits of beef strip steaks from young (Y) ^2^ and old (O) ^3^ grain-fed beef carcasses of varying marbling degrees ^4^.

Treatment	Tenderness	Juiciness	Flavor Intensity	Flavor Liking	Overall Liking
SLAB-Y	72.2 ^a^	71.3 ^a^	64.6 ^a^	65.3 ^a^	69.0 ^a^
MD-Y	69.5 ^ab^	70.9 ^a^	61.6 ^ab^	62.6 ^ab^	67.5 ^ab^
MT-Y	65.7 ^bc^	63.2 ^bc^	56.8 ^bcd^	56.2 ^bcde^	61.2 ^cd^
SM-Y	58.2 ^def^	55.2 ^de^	53.1 ^cde^	51.9 ^def^	55.0 ^def^
SL-Y	57.6 ^def^	53.0 ^de^	52.6 ^de^	51.0 ^ef^	54.8 ^ef^
SLAB-O	62.2 ^cd^	66.1 ^ab^	58.9 ^abc^	58.8 ^abc^	62.4 ^bc^
MD-O	60.4 ^cde^	64.5 ^b^	58.3 ^bcd^	57.6 ^bcd^	59.8 ^cde^
MT-O	55.2 ^efg^	58.3 ^cd^	55.4 ^bcde^	52.7 ^cdef^	55.2 ^def^
SM-O	53.4 ^fg^	55.2 ^de^	52.6 ^de^	51.5 ^def^	53.1 ^f^
SL-O	50.7 ^g^	50.4 ^e^	49.5 ^e^	47.2 ^f^	50.1 ^f^
SL-O	50.7 ^g^	50.4 ^e^	49.5 ^e^	47.2 ^f^	50.1 ^f^
SEM ^5^	1.37	1.37	1.38	1.46	1.40
*p*-value	<0.01	<0.01	<0.01	<0.01	<0.01

^a–g^ Within a column, estimated marginal means without a common superscript differ (*p* < 0.05). ^1^ Sensory scores (0 = extremely tough/dry/bland/dislike extremely; 100 = extremely tender/juicy/intense/like extremely). ^2^ Young: beef carcasses from grain-fed cattle < 30 months of age (A skeletal maturity). ^3^ Old: beef carcasses from grain-fed cattle > 30 months of age (C, D, and E skeletal maturity). ^4^ Marbling degrees: SLAB = ≥slightly abundant^00^; MD = moderate^00^ to moderate^100^; MT = modest^00^ to modest^100^; SM = small^00^ to small^100^; SL = slight^00^ to slight^100^. ^5^ SEM is the (largest) pooled standard error of least squares means.

**Table 6:** The effect of treatment group on percentage of acceptable samples for tenderness, juiciness, flavor liking, and overall liking rated by consumers (n = 320) and willingness to pay ^1^ (WTP) of beef strip steaks from young (Y) ^2^ and old (O) ^3^ fed beef carcasses of varying marbling degrees ^4^.

Treatment	Tenderness, %	Juiciness, %	Flavor Liking, %	Overall Liking, %	Willingness to Pay, $/0.45 kg
SLAB-Y	94.4 ^a^	92.5 ^a^	89.4 ^a^	92.5 ^a^	11.1 ^a^
MD-Y	91.2 ^ab^	93.7 ^a^	88.4 ^ab^	90.6 ^ab^	10.6 ^ab^
MT-Y	88.8 ^bc^	86.8 ^b^	78.1 ^cde^	83.1 ^cd^	8.8 ^bc^
SM-Y	82.8 ^de^	74.3 ^ef^	75.3 ^def^	77.8 ^de^	7.9 ^cde^
SL-Y	81.6 ^de^	73.7 ^ef^	71.9 ^ef^	79.1 ^de^	7.9 ^cde^
SLAB-O	86.2 ^c^	91.9 ^a^	83.4 ^bc^	87.2 ^bc^	9.4 ^bc^
MD-O	81.2 ^de^	85.6 ^bc^	80.6 ^cd^	82.2 ^cd^	9.1 ^bc^
MT-O	77.2 ^ef^	80.6 ^cd^	76.6 ^def^	77.5 ^de^	7.7 ^de^
SM-O	74.4 ^fg^	77.2 ^de^	73.1 ^ef^	74.7 ^ef^	7.5 ^de^
SL-O	70.3 ^g^	69.1 ^f^	70.9 ^f^	68.8 ^f^	6.9 ^e^
SEM ^5^	2.00	2.00	2.00	2.40	0.36
*p*-value	<0.01	<0.01	<0.01	<0.01	<0.01

^a–g^ Within a column, estimated marginal means without a common superscript differ (*p* < 0.05). ^1^ Sensory scores (0 = extremely tough/dry/bland/dislike extremely; 100 = extremely tender/juicy/intense/like extremely). ^2^ Young: beef carcasses from grain-fed cattle < 30 months of age (A skeletal maturity). ^3^ Old: beef carcasses from grain-fed cattle > 30 months of age (C, D, and E skeletal maturity). ^4^ Marbling degrees: SLAB = ≥slightly abundant^00^; MD = moderate^00^ to moderate^100^; MT = modest^00^ to modest^100^; SM = small^00^ to small^100^; SL = slight^00^ to slight^100^. ^5^ SEM is the (largest) pooled standard error of least squares means.

**Table 7:** Pearson correlation coefficients among consumer palatability traits, proximate composition, slice shear force (SSF), and carcass characteristics.

Attribute	Overall Liking	Tenderness	Juiciness	Flavor Liking	SSF	Marbling Score	Fat Thickness	Fat	Moisture
Tenderness	0.80 *								
Juiciness	0.70 *	0.75 *							
Flavor Liking	0.88 *	0.72 *	0.68 *						
SSF	−0.12	−0.17	−0.10	−0.13 *					
Marbling Score	0.32 *	0.33 *	0.39 *	0.33 *	−0.09				
Fat Thickness	0.21 *	0.25 *	0.22 *	0.22 *	−0.01	0.39 *			
Fat	0.34 *	0.36 *	0.39 *	0.32 *	−0.14 *	0.83 *	0.46 *		
Moisture	−0.32 *	−0.35 *	−0.37 *	−0.30 *	0.14 *	−0.79 *	−0.46 *	−0.95 *	
Protein	−0.01	0.07	−0.06	−0.05	0.05	−0.13	0.02	−0.12	0.10

* Correlation coefficient differs from 0 (*p* < 0.01).

## Data Availability

The original contributions presented in the study are included in the article, further inquiries can be directed to the corresponding author.
